# Supplementary material for: Heat adaptation of phage T7 under an extended genetic code
Source: Virus Evol. 2021 Dec 1;7(2):veab100. doi: 10.1093/ve/veab100 (PMC8923235; doi:10.1093/ve/veab100)
Supplement: veab100_Supp [file veab100_supp.zip › Sequencing_Analysis.zip!/Sequencing_Analysis/S1/evidence/JC_41.html]

BRESEQ :: Evidence


| New junction evidence | | | | | | | | | | | |
| --- | --- | --- | --- | --- | --- | --- | --- | --- | --- | --- | --- |
|  | | seq id | position | reads (cov) | reads (cov) | score | skew | freq | annotation | gene | product |
| \* | ? | NC\_001604.1 | 295 = | 5273 (1.260) | 27 (0.010) | 15/560 | 40.5 | 0.54% | intergenic (–/–) | *–/–* | –/– |
|  | ? | NC\_001604.1 | = 281 | 4936 (1.260) |  |  |  |  | intergenic (–/–) | *–/–* | –/– |
| Rejected: Coverage evenness skew score above cutoff. | | | | | | | | | | | |
| Rejected: Frequency below/above cutoff threshold. | | | | | | | | | | | |

Only 100 of 605 total aligned reads displayed.  

`TACTGCATGTTACTTCATGTTGTCAATACCTGTTTTTCGTGCGACTTATCAGGCTGTCTACTTATCCGGTTGACCCCGGTGTCGATTGGGATGGCTATTCGCCGTGTCCCTCTCGATGGCTGTAAGTATCCTATAGGTTAGACTTTAAGTCAATACTCTTTTTGATAAATTTTAAATTAATCTTTTAAGTCTCTTTAAGTTGTCTCTCCTTATAGTGAGTTGTATTAATAAAGACAATGATTAAAGGTTATCACTTTATGGCTAACAACAAGGCGTCTTTAGGTCTGGTCTTTATGTAG‑TGTCTTTAGGTCTGGTCTTTA‑‑‑‑‑‑‑‑‑‑‑‑‑‑‑‑‑‑‑‑‑‑‑‑‑‑‑‑‑‑‑‑‑‑‑‑‑‑‑‑‑‑‑‑‑‑‑‑‑‑‑‑‑‑‑‑‑‑‑‑‑‑‑‑‑‑‑‑‑‑‑‑‑‑‑‑‑‑‑‑‑‑‑‑‑‑‑‑‑‑‑‑‑‑‑‑‑‑‑‑‑‑‑‑‑‑‑‑‑‑‑‑‑‑‑‑‑‑‑‑‑‑‑‑‑‑‑‑‑‑‑‑‑‑‑‑‑‑‑‑‑‑‑‑‑‑‑‑‑‑‑‑‑‑‑‑‑‑‑‑‑‑‑‑‑‑‑‑‑‑‑‑‑‑‑‑‑‑‑‑‑‑‑‑‑‑‑‑‑‑‑‑‑‑‑‑‑‑‑‑‑‑‑‑‑‑‑‑‑‑‑‑‑‑‑‑‑‑‑‑‑‑‑‑‑‑‑‑‑‑‑‑‑‑‑‑‑‑‑‑‑‑‑‑‑‑‑‑‑‑‑‑‑‑‑‑‑‑‑‑‑‑‑‑‑‑‑‑‑‑‑‑‑‑‑‑‑‑‑‑‑‑‑‑‑‑‑‑‑‑‑‑‑‑  <  NC_001604.1/614‑295`  
`‑‑‑‑‑‑‑‑‑‑‑‑‑‑‑‑‑‑‑‑‑‑‑‑‑‑‑‑‑‑‑‑‑‑‑‑‑‑‑‑‑‑‑‑‑‑‑‑‑‑‑‑‑‑‑‑‑‑‑‑‑‑‑‑‑‑‑‑‑‑‑‑‑‑‑‑‑‑‑‑‑‑‑‑‑‑‑‑‑‑‑‑‑‑‑‑‑‑‑‑‑‑‑‑‑‑‑‑‑‑‑‑‑‑‑‑‑‑‑‑‑‑‑‑‑‑‑‑‑‑‑‑‑‑‑‑‑‑‑‑‑‑‑‑‑‑‑‑‑‑‑‑‑‑‑‑‑‑‑‑‑‑‑‑‑‑‑‑‑‑‑‑‑‑‑‑‑‑‑‑‑‑‑‑‑‑‑‑‑‑‑‑‑‑‑‑‑‑‑‑‑‑‑‑‑‑‑‑‑‑‑‑‑‑‑‑‑‑‑‑‑‑‑‑‑‑‑‑‑‑‑‑‑‑‑‑‑‑‑‑‑‑‑‑‑‑‑‑‑‑‑‑‑‑‑‑‑‑‑‑‑‑‑‑‑‑‑‑‑‑‑‑‑‑‑‑‑‑‑‑‑‑‑‑‑‑‑‑‑‑‑‑‑‑‑‑‑‑‑‑‑gtctttaggtctggtctttaTGTTTAAACTTTAAGATAGGCGTTGACTTGATGGGTCTTTAGGTGTAGGCTTTAGGTGTTGGCTTTAGGATGGACGTTAGGAGGTGACTTTAGGAGGATACTTTAGGAGACTGTAACAGATAGGGACACAGAGAGACACTCAAGGTAACACCCAAAGACAAACCCAAGGGTCATCCCCAACCCTTAGGGAACCCTCAAGGTCAACCGAAGGTTGACTTTAGGTGATTGGCTGGGCTTTAGGTACCCCCTATGGGGGAACTTTAGGTCCGTACACTGTGAGA‑‑‑‑‑‑‑‑‑‑‑‑‑  <  NC_001604.1/281‑1`  

                                                                                                                                                                                                                                                                                                                                                                                                                                                                                                                                                                                                                                         
`acagGCATGTTACTTCATGTTGTCAATACCTGTTTTTCGTGCGACTTATCAGGCTGTCTACTTATCCGGTTGACCCCGGTGTCGATTGGGATGGCTATTCGCCGTGTCCCTCTCGATGGCTGTAAGTATCCTATAGGTTAGACTTTAAGTCAATACTCTTTTTGATAAATTTTAAATTAATCTTTTAAGTCTCTTTAAGTTGTCTCTCCTTATAGTGAGTTGTATTAATAAAGACAATGATTAAAGGTTATCACTTTATGGCTAACAACAAGGCGTCTTTAGGTCTGGTCTTTATGTAG‑TG                                                                                                                                                                                                                                                                                                                           <  1:443269/297‑1`  
`TACTGCATGTTACTTCATGTAGTCAATACCAGTTTTTCGTTCGACTTATAAGGTGGTTTACTTATCCGGTTGAACCCGGTGTTGATTTGGATGGCTATTTGCCGTGTCCCTCTTGATGGCTGTAAGTATCCTATTGGTTAGGTTGTAAGTCAATACTCTTTTTGATAAATTTTAAATTAATCTTTTAAGTCTCTTTAAGTTGTCTCTCCTTATAGTGAGTTGTAGTAATAAAGACAATGATTAAAGGTTATCACTTTATGGCTAACAACAAGGCGTCTTTAGGTCTGGTCTTTATGTAG‑TG                                                                                                                                                                                                                                                                                                                           <  1:668942/301‑1`  
 `taagagatagACTTCATGTTGTCAATACCTGTTTTTCGTGCGACTTATCAGGCTGTCTACTTATCCGGTTGACCCCGGTGTCGATTGGGATGGCTATTCGCCGTGTCCCTCTCGATGGCTGTAAGTATCCTATAGGTTAGACTTTAAGTCAATACTCTTTTTGATAAATTTTAAATTAATCTTTTAAGTCTCTTTAAGTTGTCTCTCCTTATAGTGAGTTGTATTAATAAAGACAATGATTAAAGGTTATCACTTTATGGCTAACAACAAGGCGTCTTTAGGTCTGTTCTTTATGTAG‑TGT                                                                                                                                                                                                                                                                                                                          <  1:237982/291‑1`  
 `tCTGCATGTTACTTCATGTTGTCAATACCTGTTTTTCGTGCGACTTATCAGGCTGTCTACTTATCCGGTTGACCCCGGTGTCGATTGGGATGGCTATTCGCCGTGTCCCTCTCGATGGCTGTAAGTATCCTATAGGTTAGACTTTAAGTCAATACTCTTTTTGATAAATTTTAAATTAATCTTTTAAGTCTCTTTAAGTTGTCTCTCCTTATAGTGAGTTGTATTAATAAAGACAATGATTAAAGGTTATCACTTTATGGCTAACAACAAGGCGTCTTTAGGTCTGGTCTTTATGTAG‑TGT                                                                                                                                                                                                                                                                                                                          <  1:546696/300‑1`  
 `gCTGCATGTTACTTCATGTTGTCAATACCTGTTTTTCGTGCGACTTATCAGGCTGTCTACTTATCCGGTTGACCCCGGTGTCGATTGGGATGGCTATTCGCCGTGTCCCTCTCGATGGCTGTAAGTATCCTATAGGTTAGACTTTAAGTCAATACTCTTTTTGATAAATTTTAAATTAATCTTTTAAGTCTCTTTAAGTTGTCTCTCCTTATAGTGAGTTGTATTAATAAAGACAATGATTAAAGGTTATCACTTTATGGCTAACAACAAGGCGTCTTTAGGTCTGGTCTTTATGTAG‑TGT                                                                                                                                                                                                                                                                                                                          <  1:129851/300‑1`  
 `caTGCATGTTACTTCATGTTGTCAATACCTGTTTTTCGTGCGACTTATCAGGCTGTCTACTTATCCGGTTGACCCCGGTGTCGATTGGGATGGCTATTCGCCGTGTCCCTCTCGATGGCTGTAAGTATCCTATAGGTTAGACTTTAAGTCAATACTCTTTTTGATAAATTTTAAATTAATCTTTTAAGTCTCTTTAAGTTGTCTCTCCTTATAGTGAGTTGTATTAATAAAGACAATGATTAAAGGTTATCACTTTATGGCTAACAACAAGGCGTCTTTAGGTCTGGTCTTTATGTAG‑TGT                                                                                                                                                                                                                                                                                                                          <  1:403591/299‑1`  
 `ACTGCATGTTACTTCATGTTGTCAATACCTGTTTTTCGTGCGACTTATCAGGCTGTCTACTTATCCGGTTGACCCCGGTGTCGATTGGGATGGCTATTCGCCGTGTCCCTCTCGATGGCTGTAAGTATCCTATAGGTTAGACTTTAAGTCAATACTCTTTTTGATAAATTTTAAATTAATCTTTTAAGTCTCTTTAAGTTGTCTCTCCTTATAGTGAGTTGTATTAATAAAGACAATGATTAAAGGTTATCACTTTATGGCTAACAACAAGGCGACTTTAGGTCTGGTCTTTATGTAG‑TGT                                                                                                                                                                                                                                                                                                                          >  1:408938/1‑301`  
 `ACTGCATGTTACGTCATGTTGTCAATACCTGTTTTTCGTGCTACTTATCAGGCTGTCTACTTATCCGGTTGACCCCGGTGTCGATTGGGATGGCTATTCGCCGTGTCCCTCTCGATGGCTGTAAGTATCCTATAGGTTAGACTTTAAGTCAATACTCTTTTTGATAAATTTTAAATTAATCTTTTAAGTCTCTTTAAGTTGTCTCTCCTTATAGTGAGTTGTATTAATAAAGACAATGATTAAAGGTTATCACTTTATGGCTAACAACAAGGCGTCTTTAGGTCTGGTCTTTATGTAG‑TGT                                                                                                                                                                                                                                                                                                                          <  1:52576/301‑1`  
  `CTGCATGTTACTTCATGTTGTCAATACCTGTTTTTCGTGCGACTTATCAGGCTGTCTACTTATCCGGTTGACCCCGGTGTCGATTGGGATGGCTATTCGCCGTGTCCCTCTCGATGGCTGTAAGTATCCTATAGGTTAGACTTTAAGTCAATACTCTTTTTGATAAATTTTAAATTAATCTTTTAAGTCTCTTTAAGTTGTCTCTCCTTATAGTGAGTTGTATTAATAAAGACAATGATTAAAGGTTATCACTTTATGGCTACCAACAAGGCGTCTTTAGGTCTGGTCTTTATGTAG‑TGTC                                                                                                                                                                                                                                                                                                                         <  1:230619/301‑1`  
  `CTGCATGTTACTTCATGTTGTCAATACCTGTTTTTCGTGCGACTTATCAGGCTGTCTACTTATCCGGTTGACCCCGGTGTCGATTGGGATGGCTATTCGCCGTGTCCCTCTCGATGGCTGTAAGTATCCTATAGGTTAGACTTTAAGTCAATACTCTTTTTGATAAATTTTAAATTAATCTTTTAAGTCTCTTTAAGTTGTCTCTCCTTATAGTGAGTTGTATTAATAAAGACAATGATTAAAGGTTATCACTTTATGGCTAACAACAAGGCGTCTTTAGGTCTGGTCTTTATGTAG‑TGTC                                                                                                                                                                                                                                                                                                                         <  1:178645/301‑1`  
    `GCATGTTACTTCATGTTGTCAATACCTGTTTTTCGTGCGACTTATCAGGCTGTCTACTTATCCGGTTGACCCCGGTGTCGATTGGGATGGCTATTCGCCGTGTCCCTCTCGATGGCTGTCAGTATCCTATAGGTTAGACTTTAAGTCAATACTCTTTTTGATAACTTTTAAATTAATCTTTTAAGTCTCTTTAAGTTGTCTCTCCTTATAGTGAGTTGTATTAATAAAGACAATGATTAAAGGTTATCACTTTATGGCTACCAACAGGGCGTCTTTAGGTCTGGTCTTTATGTAG‑TGTCTT                                                                                                                                                                                                                                                                                                                       >  1:85084/1‑301`  
    `GCATGTTACTTCATGTTGTCAATACCTGTTTTTCGTGCGACTTATCAGGCTGTCTACTTATCCGGTTGACCCCGGTGTCGATTGGGATGGCTATTCGCCGTGTCCCTCTCGATGGCTGTAAGTATCCTATAGGTTAGACTTTAAGTCAATACTCTTTTTGATAAATTTTAAATTAATCTTTTAAGTCTCTTTAAGTTGTCTCTCCTTATAGTGAGTTGTATTAATAAAGACAATGATTAAAGGTTATCACTTTATGGCTAACAACAAGGCGCCTTTAGGTCTGGTCTTTATGTAG‑TGTCTT                                                                                                                                                                                                                                                                                                                       >  1:416797/1‑301`  
      `ATGTTACTTCATGTTGTCTATACCTGTTTTTCGTGCGACTTATCAGGCTGTCTACTTATCAGGTTGACCCCGGTGTAGTTTGGGATGGCTATTCGCCGTGTCCCTCTCGATGGCTGTAAGTATCCTATAGGTTGGACTTTACGTCAATACTCTCTTTGATAAATTTTAGATTAATCTTTTAAGTCTCTTTAAGTTGTCTCTCCTTATAGTGAGTTGTATTAATAAAGACTATTATTAAATGTTATCACTTTATGGCTAACAACAAGGCGTCTTTAGTTCTGGTCTTTATTTTG‑TGTCTTTt                                                                                                                                                                                                                                                                                                                     >  1:198389/1‑300`  
      `ATGTTACTTCATGTTGTCAATACCTGTTTTTCGTGCGACTTATCAGGCTGTCTACTTATCCGGTTGACCCCGGTGTCGATTGGGATGGCTATTCGCCGTGTCCCTCTCGATGGCTGTAAGTATCCTATAGGTTAGACTTTAAGTCAATACTCTTTTTGATAAATTTTAAATTAATCTTTTAAGTCTCTTTAAGTTGTCTCTCCTTATAGTGAGTTGTATTAATAAAGACAATGATTAAAGGTTATCACTTTATGGCTAACAACAAGGCGTCTTTAGGTCTGGTCTTTATGTAGCTGTCTct                                                                                                                                                                                                                                                                                                                      >  1:590862/1‑299`  
      `ATGTTACTTCATGTTGTCAATACCTGTTTTTCGTGCGACTTATCAGGCTGTCTACTTATCCGGTTGACCCCGGTGTCGATTGGGATGGCTATTCGCCGTGTCCCTCTCGATGGCTGTAAGTATCCTATAGGTTAGACTTTAAGTCAATACTCTTTTTGATAAATTTTAAATTAATCTTTAAAGTCTCTTTAAGTTGTCTCTCCTTATAGTGAGTTGTATTAATAAAGACAATGATTAAAGGTTATCACTTTATGGCTAACAACAAGGCGTCTTTAGGTCTGGTCTTTATGTAG‑TGTCTTTt                                                                                                                                                                                                                                                                                                                     >  1:533693/1‑300`  
       `gGTTACTTCATGTTGTCAATACCTGTTTGTCGTGCGAATTATCAGGCTGTCTACTTATCCGGTTGACCCCGGTGTCGATTGGGATGGCTATTCGCCGTGTCCATCTCGATGGCTGTAAGTATCCTATAGGTTAGACTTTAAGTCAATACTCTTTTTGATAAATTTTAAATTAATCTTTTAAGTCTCTTTAAGTTGTCTCTCCTTATAGTGAGTTGTATTAATAAAGACAATGATTAAAGGTTATCACTTTATGGCTAACAACAAGGCGTCTTTAGGTCTGGTCTTTATGTAG‑TGTCTTTAG                                                                                                                                                                                                                                                                                                                    <  1:367968/300‑1`  
       `TGTTACTTCATGTTGTCAATACCTGTTTTTCGTGCGACTTATCAGGCTGTCTACTTATCCGGTTGACCCCGGTGTCGATTGGGATGGCTATTCGCCGTGTCCCTCTCGATGGCTGTAAGTATCCTATAGGTTAGACTTTAAGTCAATACTCTTTTTGATAAATTTTAAATTAATCTTTTAAGTCTCTTTAAGTTGTCTCTCCTTATAGTGAGTTGTATTAATAAAGACAATGATTCAAGGTTATCACTTTATGGCTAACAACAAGGCGTCTTTAGGTCTGGTCTTTATGTAG‑TGTCTTTAG                                                                                                                                                                                                                                                                                                                    >  1:531469/1‑301`  
        `tTTACTTCATTTTGTCAATACCTGTTTTTCGTGCGACTTATAAGGCTGTCTACTTATACGGTTGACCCCGGTGTCGATTGGGATGGCTATTCGCCGTGTCCCTCTCGATGGCTGTAAGTATCCTATAGGTTAGACTTTAAGTCAATACTCTTTTTGATAAATTTTAAATTAATCTTTTAAGTCTCTTTAAGTTGTCTCTCCTTATAGTGAGTTGTATTAATAAAGACAATGATTAAAGGTTATCACTTTATGGCTAACAACAAGGCGTCTTTAGGTCTGGTCTTTATGTAG‑TGTCTTTAGG                                                                                                                                                                                                                                                                                                                   <  1:376608/300‑1`  
        `tTTACTTCATGTTGTCAATTCCTGTTTTTCGTGCGACTTATCAGGCTGTCTACTTATCCGGTTGACCCCGGTGTCGATTGGGATGGCTATTCGCCGTGTCCCTCTCGATGGCTGTAAGTATCCTATAGGTTAGACTTTAAGTCAATACTCTTTTTGATAAATTTTAAATTAATCTTTTAAGTCTCTTTAAGTTGTCTCTCCTTATAGTGAGTTGTATTAATAAAGACAATGATTAAAGGTTATCACTTTATGGCTAACAACAAGGCGTCTTTAGGTCTGGTCTTTATGTAG‑TGTCTTTAGG                                                                                                                                                                                                                                                                                                                   <  1:625325/300‑1`  
        `tTTACTTCATGTTGTCAATACCTGTTTTTCGTGCGACTTATCAGGCTGTCTACTTATCCGGTTGACCCCGGTGTCGATTGGGATGGCTATTCGCCTTGTCCCTCTCGATGGCTGTAAGTATCCTATAGGTTAGACTTTAAGTCAATACTCTTTTTGATAAATTTTAAATTAATCTTTTAAGTCTCTTTAAGTTGTCTCTCCTTATAGTGAGTTGTATTAATAAAGACAATGATTAAAGGTTATCACTTTATGGCTAACAACAAGGCGTCTTTAGGTCTGGTCTTTATGTAG‑TGTCTTTAGG                                                                                                                                                                                                                                                                                                                   <  1:92629/300‑1`  
        `tTTACTTCATGTTGTCAATACCTGTTTTTCGTGCGACTTATCAGGCTGTCTACTTATCCGGTTGACCCCGGTGTCGATTGGGATGGCTATTCGCCGTGTCCCTCTCGATGGCTGTAAGTATCCTATAGGTTAGACTTTAAGTCAATACTCTTTTTGATAAATTTTAAATTAATCTTTTAAGTCTCTTTAAGTTGTCTCTCCTTATAGTGAGTTGTATTAATAAAGACAATGATTAAAGGTTATCACTTTATGGCTAACAACAAGGCGTCTTTAGGTCTGGTCTTTATGTAG‑TGTCTTTAGG                                                                                                                                                                                                                                                                                                                   <  1:218380/300‑1`  
        `GTTACTTCATGTTGTCAATACCTGTTTTTCGTGCGACTTATCAGGCTGTCTGCTTATCCGGTTGACCCCGGTGTCGATTGGGATGGCTATTCGCCGTGTCCCTCTCGATGGCTGTAAGTATCCTATAGGTTAGACTTTAAGTCAATACTCTTTTTGATAAATTTTAAATTAATCTTTTAAGTCTCTTTAAGTTGTCTCTCCTTATAGTGAGTTGTATTAATAAAGACAATGATTAAAGGTTATCACTTTATGGCTAACAACAAGGCGTCTTTAGGTCTGGTCTTTATGTAG‑TGTCTTTAGG                                                                                                                                                                                                                                                                                                                   <  1:79106/301‑1`  
        `GTTACTTCATGTTGTCAATACCTGTTTTTCGTGCGACTTATCAGGCTGTCTACTTATCCGGTTGACCCCGGTGTCGATTGGGATGGCTATTCGCCGTGTCCCTCTCGATGGCTGTAAGTATCCTATAGGTTAGACTTTAAGTCAATACTCTTTTTGATAAATTTTAAATTAATCTTTTAAGTCTCTTTAAGTTGTCTCTCCTTATAGTGAGTTGTATTAATAAAGACAATGATTAAAGGTTATCACTTTATGGCTAACAACAAGGCGTCTTTAGGTCTGGTCTTTATGTAG‑TGTCTTTAGG                                                                                                                                                                                                                                                                                                                   <  1:432783/301‑1`  
         `TTACTTCATGTTGTCAATACCTGTTTTTCGTGCGACTTATCAGGCTGTCTACTTATCCGGTTTACCCCGGTGTCGATTTGGATGGCTATTCGCCGTGTCCCTCTCTATGGCTGTCAGTATCCTATAGGTTAGACTTTAAGTCAATACCCTTTTTGATAAATTTTAAATTATTCTTTTAAGTCTCTTTAATTTGTCGCTCCTTATATTGAGTTGTATTAATAAAGACAATTATTAAAGGTTATCACTTTATGTCTAACAACAAGTCCTTTTTAGTTCTGTTCTTTATGTAT‑TGTCTTTAGGT                                                                                                                                                                                                                                                                                                                  >  1:619383/1‑301`  
         `TTACTTCATGTTGTCAATACCTGTTGTTCGTGCGACTTATCAGGCTGTATACTGAGCCGGTTGACACCGG‑GTAGATTGGGATGGCAATTAGCCGTGTCCCTCTCGATGGCTGTAAGTATCCTATAGGATAGACTTTAAGTCAATACGCTTTTTGATAAATTTTAAATTAATATTTTAAGTCTATTTAAGTTGTCTCGCCTGATAGTGAGTTGTATTAATAAAGACAATGATTAAAGGTTATCACTTTAGGGCTAACAACAAGGCGTCTTTAGGTCTGGTCTTTATGTAG‑TGTCTTTAGGTC                                                                                                                                                                                                                                                                                                                 <  1:285016/301‑1`  
          `tctTTCATGTTTTCCATCCCTGTTTTTCGTGCGACTTATCAGGCTGTCTACTTATCCGGTTGACCCCGGTGTCGATTGGGATGGCTATTCGCCGTGTCCCTCTCGATGGCTGTAAGTATCATATAGGTTTGACTTTAAGTCAATACTCTTTTTGATAAATTTTAAATTTATCTTTTAAGTCTCTTTAAGTTGTCTCTCCTTATAGTGAGTTGTATTAATAAAGACAATTATTAAAGGTTATCACTTTATGGCTAACAACAAGGCGTCTTTAGGTCTGGTCTTTATGTAG‑TGTCTTTAGGTC                                                                                                                                                                                                                                                                                                                 <  1:500616/298‑1`  
          `tcaTTCATGTTGTCAATACCTGTTTTTCGTGCGACTTATCAGGCTGTCTACTTATCCGGTTGACCCCGGTGTCGATTGGGATGGCTATTCGCCGTGTCCCTCTCGATGGCTGTAAGTATCCTATAGGTTAGACTTTAAGTCAATACTCTTTTTGATAAATTTTAAATTAATCTTTTAAGTCTCTTTAAGTTGTCTCTCCTTATAGTGAGTTGTATTAATAAAGACAATGATTAAAGGTTATCACTTTATGGCTAACAACAAGGCGTCTTTAGGTCTGGTCTTTATGTAG‑TGTCTTTAGGTC                                                                                                                                                                                                                                                                                                                 <  1:522296/298‑1`  
          `gACTTCATGTTGTCAATACCTGTTTTTCGTGCGACTTATCAGGCTGTCTACTTATCCGGTTGACCCCGGTGTCGATTGGGATGGCTATTCGCCGTGTCCCTCTCGATGGCTGTAAGTATCCTATAGGTTAGACTTTAAGTCAATACTCTTTTTGATAAATTTTAAATTAATCTTTTAAGTCTCTTTAAGTTGTCTCTCCTTATAGTGAGTTGTATTAATAAAGACAATGATTAAAGGTTATCACTTTATGGCTAACAACAAGGCGTCTTTAGGTCTGGTCTTTATGTAG‑TGTCTTTAGGTC                                                                                                                                                                                                                                                                                                                 <  1:514561/300‑1`  
          `ataataaacaggtgAATACCTGTTTTTCGTGTGGCTTATCAGGCTGTCTACTTATCCGGTTGACCCCGGTTTCGATTGGAATGGCTATGCACCGTGTCCCTCTCGATGGCTGTAAGTATCCTATAGGTTAGGCTTTAAGTCAATACTCTTTTTGATATATTTTAAATTAATCTTTTAAGTCTCTTTAAGTTGTCTCTCATTATAGTGAGTTGTATTAATAAAGACAATGATTAAAGGTTATCACTTTATGGCTAACAACAAGGCGTCTTTAGGTCTGGTCTTTATGTAG‑TGTCTTTAGGTC                                                                                                                                                                                                                                                                                                                 <  1:537359/287‑1`  
          `TACTTCATGTTGTCAATACCTGTTTTTCGTGCGACTTATCCGGCTGTCTACTTATCCGGTTGACCCCGGTGTCGAGTGGAATGGCGATTCGCCGTGTCCCTCTCGATGGCTGTAAGTATCCTATAGGTTAGACTTTAAGTCACGACTCTTTTTGATAAATTTTAAATTAATATTTTAAGTCTCTTTAAGTTGTCTCTCCTTATAGTGAGTTGTATTAATAAAGACAATGATTAAAGGTTATCACTTAATGGCTAACAACAAGGCGTCTTAAGGTCTGGTATTTTTGTAG‑AGTCTATAGGTg                                                                                                                                                                                                                                                                                                                 >  1:316186/1‑300`  
          `TACTTCATGTTGTCAATACCTGTTTTTCGTGCGACTTATCAGGCTGTCTACTTATCCGGTTGACCCCGGTGTCGATTGGTATGGCTATTCGCCGTGTCCCTCTCGATGGCTGTAAGTATCCTATAGGTTAGACTTTAAGTCAATACTCTTTTTGATAAATTTTAAATTAATCTTTTAAGTCTCTTTAAGTTTTCTCTCCTTATAGTGAGTTGTATTAATAGAGACAATGATTAAAGGTTATCACTTTCTGTCTACCAACAAGGCGTCTTTAGGTCTGGTCTTTTTGTAT‑TTTCTTTAGGTt                                                                                                                                                                                                                                                                                                                 >  1:37435/1‑300`  
          `TACTTCATGTTGTCAATACCTGTTTTTCGTGCGACTTATCAGGCTGTCTACTTATCCGGTTGACCCCGGTGTCGATTGGGATGGCTATTCGCCGTGTCCCTCTCGATGGCTGTAAGTATCCTATAGGTTAGACTTTAAGTCAATACTCTTTTTGATAAATTTTAAATTAATCTTTTAAGTCTCTTTACGTTGTCTTTCCTTATAGGGAGTTATATTAATAAAGACATAGATTAAAGGTTTTCACTTTATGGCTAACAACAAGGCGTCTTTTGGTCTGGTCTTTATGTAG‑TGTCTTTAGGct                                                                                                                                                                                                                                                                                                                 >  1:142334/1‑299`  
          `TACTTCATGTTGTCAATACCTGTTTTTCGTGCGACTTATCAGGCTGTCTACTTATCCGGTTGACCCCGGTGTCGATTGGGATGGCTATTCGCCGTGTCCCTCTCGATGGCTGTAAGTATCCTATAGGTTAGACTTTAAGTCAATACTCTTTTTGATAAATTTTAAATTAATCTTTTAAGTCTCTTTAAGTTGTCTCTCCTTATAGTGAGTTGTATTAATAAAGACAATGATTAAAGGTTATCACTTTATGGCTAACAACAAGGCGTCTTTAGGTCTGGTCTTTATGTAG‑TGTCTTTAGGTt                                                                                                                                                                                                                                                                                                                 >  1:498500/1‑300`  
          `TACTTCATGTTGTCAATACCTGTTTTTCGTGCGACTTATCAGGCTGTCTACTTATCCGGTTGACCCCGGTGTCGATTGGGATGGCTATTCGCCGTGTCCCTCTCGATGGCTGTAAGTATCCTATAGGTTAGACTTTAAGTCAATACTCTTTTTGATAAATTTTAAATTAATCTTTTAAGTCTCTTTAAGTTGTCTCTCCTTATAGTGAGTTGTATTAATAAAGACAATGATTAAAGGTTATCACTTTATGGCTAACAACAAGGCGTCTTTAGGTCTGGTCTTTATGTAG‑TGTCTTTAGGTt                                                                                                                                                                                                                                                                                                                 >  1:207731/1‑300`  
          `TACTTCATGTTGTCAATACCTGTTTTTCGTGCGACTTATCAGGCTGTCTACTTATCCGGTTGACCCCGGTGTCGATTGGGATGGCTATTCGCCGTGTCCCTCTCGATGGCTGTAAGTATCCTATAGGTTAGACTTTAAGTCAATACTCTTTTTGATAAATTTTAAATTAATCTTTTAAGTCTCTTTAAGTTGTCTCTCCTTATAGTGAGTTGTATTAATAAAGACAATGATTAAAGGTTATCACTTTATGGCTAACAACAAGGCGTCTTTAGGTCTGGTCTTTATGTAG‑TGTCTTTAGGTC                                                                                                                                                                                                                                                                                                                 <  1:235542/301‑1`  
          `TACTTCATGTTGTCAATACCTGTTTTTCGTGCGACTTATCAGGCTGTCTACTTATCCGGTTGACCCCGGTGTCGATTGGGATGGCTATTCGCCGTGTCCCTCTCGATGGCTGTAAGTATCCTATAGGTTAGACTTTAAGTCAATACTCTTTTTGATAAATTTTAAATTAATCTTTTAAGTCTCTTTAAGTTGTCTCTCCTTATAGTGAGTTGTATTAATAAAGACAATGATTAAAGGTTATCACTTTATGGCTAACAACAAGGCGTCTTTAGGTCTGGTCTTTATGTAG‑TGTCTTTAGGTC                                                                                                                                                                                                                                                                                                                 <  1:552059/301‑1`  
          `TACTTCATGTTGTCAATACCTGTTTTTCGTGCGACTTATCAGGCTGTCTACTTATCCGGTTGACCCCGGTGTCGATTGGGATGGCTATTCGCCGTGTCCCTCTCGATGGCTGTAAGTATCCTATAGGTTAGACTTTAAGTCAATACTCTTTTTGATAAATTTTAAATTAATCTTTTAAGTCTCTTTAAGTTGTCTCTCCTTATAGTGAGTTGTATTAATAAAGACAATGATTAAAGGTTATCACTTTATGGCTAACAACAAGGCGTCTTTAGGTCTGGTCTTTATGTAG‑TGTCTTTAGGTC                                                                                                                                                                                                                                                                                                                 <  1:278546/301‑1`  
          `TACTTCATGTTGTCAATACCTGTTTTTCGTGCGACTTATCAGGCTGTCTACTTATCCGGTTGACCCCGGTGTCGAGTGGGATGGCTATTCGCCGTGTCCCTCTCGATGGCTGTAAGTATCCTATAGGTTAGACTTTAAGTCAATACTCTTTTTGATAAATTTTAAATTAATCTTTTAAGTCTCTTTAAGTTGTCTCTCCTTATAGTGAGTTGTATTAATAAAGACAATGATTAAAGGTTATCACTTTATGGCTAACAACAAGGCGTCTTTAGGTCTGGTCTTTATGTAG‑TGTCTTTAGGTC                                                                                                                                                                                                                                                                                                                 <  1:485414/301‑1`  
          `TACTTCATGTTGTCAATACCTGTTTTTCGTGCGACTTATCAGGCTGTCTACTTATCAGGTTGACCCCGGTGTCGATTGGGATGGCTATTCGCCGTGTCCCTCTCGATGGCTGTAAGTATCCTATAGGTTAGACTTTAAGTCAATACTCTTTTTGATAAATTTTAAATTAATCTTTTAAGTCTCTTTAAGTTGTCTCTCCTTATAGTGAGTTGTATTAATAAAGACAATGATTAAAGGTTATCACTTTATGGCTAACAACAAGGCGTCTTTAGGTCTGGTCTTTATGTAG‑TGTCTTTAGGTC                                                                                                                                                                                                                                                                                                                 <  1:506716/301‑1`  
           `ACTTCATGTTGTCAATACCTGTTTTTCGTGCGACTTATCAGGCTGTCTACTTATCCGGTTGACCCCGGTGTCGATTGGGATGGCTATTCGCCGTGTCCCTCTCGATGGCTGTAAGTATCCTATAGGTTAGACTTTAAGTCAATACTCTTTTTGATAAATTTTAAATTAATCTTTTAAGTCTCTTTAAGTTGTCTCTCCTTATAGTGAGTTGTATTAATAAAGACAATGATTAAAGGTTATCACTTTATGGCTAACAACAAGGCGTCTTTAGGTCTGGTCTTTATGTAGCTGTCTcttataa                                                                                                                                                                                                                                                                                                                 >  1:336605/1‑294`  
           `ACTTCATGTTGTCAATACCTGTTTTTCGTGCGACTTATCAGGCTGTCTACTTATCCGGTTGACCCCGGTGTCGATTGGGATGGCTATTCGCCGTGTCCCTCTCGATGGCTGTAAGTATCCTATAGGTTAGACTTTAAGTCAATACTCTTTTTGATAAATTTTAAATTAATCTTTTAAGTCTCTTTAAGTTGTCTCTCCTTATAGTGAGTTGTATTAATAAAGACAATGATTAAAGGTTATCACTTTATGGCTAACAACAAGGCGTCTTTAGGTCTGGTCTTTATGTAG‑TGTCTTTAGGTCT                                                                                                                                                                                                                                                                                                                >  1:108089/1‑301`  
            `CTTCATGTTGTCAATACCTGTTTTTCGTGCGACTTATCAGGCTGTCTACTTATCCGGTTGACCCCGGTGTCGATTGGGATGGCTATTCGCCGTGTCCCTCTCGATGGCTGTAAGTATCCTATAGGTTAGACTTTAAGTCAATACTCTTTTTGATAAATTTTAAATTAATCTTTTAAGTCTCTTTAAGTTGTCTCTCCTTATAGTGAGTTGTATTAATAAAGACAATGATTAAAGGTTATCACTTTATGGCTAACAACAAGGCGTCTTTAGGTATGGTCTTTATGTAGCTGTCTcatataca                                                                                                                                                                                                                                                                                                                >  1:607626/1‑293`  
              `TCATGTTGTCAATACCTGTTATGCGTGAGAGTTGTCAGGCTGTCTACTTATCCGGTTGACCCCGGTGTCGAATGGGTTGGCTATTCGCCGTGTCGCTCTCGATGGCTGTAAGTATCCTATAGGTTAGACTTTAAGTCAATACTCTGTTTGATAAATTTTAAATTAATCTTTTAAGTCTCTTTAAGTTGTCTCTCCTTATAGTGAGTTGTATTAATAAAGACAATGATTAAAGGTTATCACTTTATGGCTATCAACAAGGCGTCTTTAGGTCTGGTCTTTATGTAG‑TGTCTTTAGGTCTGGT                                                                                                                                                                                                                                                                                                             <  1:304789/301‑1`  
               `cgtgtgcgcgtagatgtgtataagagacagTTATCAGGCTGTCTACTTATCCGGTTGACCCCGGTGTCGATTGGGATGGCGATTCGCCGTGTCCCTCTCGAGGGCTGTAAGTATCCTATAGGTTAGACTTTAAGTCAATACTCTTTTTGATAAATTTTAAATTAATCTGTTAAGTCTCTTTAAGTTGTCTCTCCTTATAGTGAGTTGTATTAATAAAGACAATGATTAAAGGTTATCACTTTATGGCTAACAACAAGGCGTCTTTAGGTCTGGTCTTTATGTAG‑TGTCTTTAGGTCTGGTC                                                                                                                                                                                                                                                                                                            <  1:111849/271‑1`  
               `CATGTTGTCAATACCTGTTTTTCGTGCGACTTATCAGGCTGTCTACTTATCCGGTTGACCCCGGTGTCGATTGGGATGGCTATTCGCCGTGTCCCTCTCGATGGCTGTAAGTATCCTATAGGTTAGACTTTAAGTCAATACTCTTTTTGATAAATTTTAAATTAATCTTTTAAGTCTCTTTAAGTTGTCTCTCCTTATAGTGAGTTGTATTAATAAAGACAATGATTAAAGGTTATCACTTTATGGCTAACAACAAGGCGTCTTTAGGTCTGGTCTTTATGTAGCTGTCTcttatacacat                                                                                                                                                                                                                                                                                                             >  1:7568/1‑290`  
               `CATGTTGTCAATACCTGTTTTTCGTGCGACTTATCAGGCTGTCTACTTATCCGGTTGACCCCGGTGACGATTGAGATGGCTATTCGCCGTGTCCCTCTCGATGGCTGTAAGTATCCTATAGGTTAGACTTTAAGTCAATACTCTTTTTGATAAATTTTAAATTAATCTTTTAAGTCTCTTTAAGTTGTCTCTCCTTATAGTGAGTTGTATTAATAAAGACAATGATTAAAGGTTATCACTTTATGGCTAACAACAAGGCGTCTTTAGGTCTGGTCTTTATGTAG‑TGTCTTTAGGTCTGGTC                                                                                                                                                                                                                                                                                                            <  1:603863/301‑1`  
               `CATGTTGTCAATACCTGTTTTTCGTGCGACTGATCAGGCTGTCTACTTATCAGGTTGACCCCGGTGTCGATTGGGATGGCTATTCGCCGTGTCCCTCTCGATGGCTGTAAGTATCCTATAGGTTAGACTTTAAGTCAATACTCTTTTTGATAAATTTTAAATTAATCTTTTAAGTCGCTTTAAGTTGTCTCTCCTTATAGTGAGTTGTATTAATAAAGACAATGATTAAAGGTTATCACTTTATGGCTAACAACAAGGCGTCTTTAGGTCTGGTCTTTATGTAG‑TGTCTTTAGGTCTGGTC                                                                                                                                                                                                                                                                                                            <  1:448492/301‑1`  
                    `TGTCAATACCTGTTTTTCGTGCGACTTATCAGGCTGTCTACTTATCCGGTTGACCCCGGTGTCGATTGGGATGGCTATTCGCCGTGTCCCTCTCGATGGCTGTAAGTATCCTATAGGTTAGACTTTAAGTCAATACTCTTTTTGATAAATTTTAAATTAATCTTTTAAGTATCTTTCAGTTGTCTCTCCTTATAGTGAGTTGTATTAATAAAGACAATGATTAAAGGTTATCACTTTATGGCTAACAACAAGGCGTATTTAGGTCTGGTTTTTTTGTAG‑TGTCTTTAGGctgtctcttata                                                                                                                                                                                                                                                                                                       >  1:194035/1‑289`  
                     `tTCAGTACCTGTTTGTCGGGCGACTTATCAGGCTGTCTACTTATCCGGTTGACCCCGGTGGCGATTGGGATGGCTATTCGCCGTGTCCCTCTCGATGGCTGTAAGTATCCTATAGGTTAGACTTTAAGTCAATACTCTTTTTGATAAATTTTAAATTAATCTTTTAAGTCTCTTTAAGTTGTCTCTCCTTATAGTGAGTTGTATTAATAAAGACAATGATTAAAGGTTATCACTTTATGGCTAACAACAAGGCGTCTTTAGGTCTGGTCTTTATGTAG‑TGTCTTTAGGTCTGGTCTTgagg                                                                                                                                                                                                                                                                                                      <  1:325228/300‑5`  
                     `tTCAATCCCTGTTTTTCGTGCGACTTATCCGGCTGTATACTTATCCGGTTGGCACCGGTGTAGATTGGGATGGCTATTCGCCGTGTCCCTCTCGATGGCTGTAAGTATCCTATAGGTTAGACTTTAAGTCAATACTCTTTTTGATAAATTTTAAATTAATCTTTTAAGTCTCTTTAAGTTGTCTCTCCTTATAGTGAGTTGTATTAATAAAGACAATGATTAAAGGTTATCACTTTATGGCTAACAACAAGGCGTCTTTAGGTCTGGTCTTTATGTAG‑TGTCTTTAGGTCTGGTCTTgagg                                                                                                                                                                                                                                                                                                      <  1:69728/300‑5`  
                           `ACCTATTTTTCGTGCGACTTATCAGGCTGTCTACTTATCCGGTTGACCCCGGTGTCGATTGGGATGGCTATTCGCCGTGTCCCTCTCGATGGCTGTAAGTATCCTATAGGTTAGACTTTAAGTCAATACTCTTTTTGGTAAATTTTAAATTAATCTTTTAAGTCTCTTTAAGTTGTCTCTCCTTATAGTGAGTTGTATTAATAAAGACAATGATTAAAGGTTATCACTTTATGGCTAACAACAAGTCGTCTTTAGGTCTGGTCTTTATGTAG‑TGctgtctcttatacacatctccgagccc                                                                                                                                                                                                                                                                                                >  1:282260/1‑274`  
                             `CTGTTTTTCGTGCGACTTATCAGGCTGTCTACTTATCCGGTTGACCCCGGTGTCGATTGGGATGGCTATTCGCCGTGTCCCTCTCGATGGCTGTAAGTATCCTATAGGTTAGACTTTAAGTCAATACTCTTTTTGATAAATTTTAAATTAATCTTTTAAGTCTCTTTAAGTTGTCTCTCCTTATAGTGAGTTGTATTAATCAAGACAATGATTAAAGGTTATCACTTTATGGCTAACAACAAGGCGTCTTTAGGTCTGGTCTTTATGTAG‑TGTCTctgtctcttttacacatctccgcgcc                                                                                                                                                                                                                                                                                              >  1:101115/1‑275`  
                                            `CTTATCAGGCTGTCTACTTATCCGGTTGACCCCGGTGTCGATTGGGATGGCTATTCGCCGTGTCCCTCTCGATGGCTGTAAGTATCCTATAGGTTAGACTTTAAGTCAATACTCTTTTTTATAAATTTTAAATTAATCTTTTAAGTCTCTTTAAGTTGTCTCTCCTTATCATCATTTGTATTAATACAGACAATGATTAAAGGTTATCACTTTATGGCCAACAACAAGGCGTCTTTAGCTCGGGTCTTCATCTAG‑TGTCTTTAGTTCTGGTCTTTATGTTTtttgttgatgctgtatcttt                                                                                                                                                                                                                                                                               >  1:520600/1‑281`  
                                            `CTTATCAGGCTGTCTACTTATCCGGTTGACCCCGGTGTCGATTGGGATGGCTATTCGCCGTGTCCCTCTCGATGGCTGTAAGTATCCTATAGGTTAGACTTTAAGTCAATACTCTTTTTGATAAATTTTAAATTAATCTTTTAAGTCTCTTTAAGTTGTCTCTCCTTATAGTGAGTTGTATTAATAAAGACAATGATTAAAGGTTATCACTTTACGGCTAACAACAAGGCGTCTTTAGGTCTGGTCTTTATGTAG‑TGTCTTTAGGTCTGGTCctgtctcttatacacatctccgagcccac                                                                                                                                                                                                                                                                               >  1:246585/1‑272`  
                                                                                                                   `attGCTTTAATTTTCCTATAGGTTAGACTTTAAGTCAATACTCTTTTTGATAAATTTTAAATTAATCTTTTAAGTCTCTTTAAGTTGTCTCTCCTTATAGTTAGTTGTATTAATAAAGACAATGATTAAAGGTTATCACTTTATGGCTAACAACAAGGCGTCTTTAGGTCTGGTCTTTATGTAG‑TGTCTTTAGGTCTTGTCTTTATGTTTAAACTTTAAGATAGGCGTTGACTTGATGGGTCTTTAGGTGTAGGCTTTAGGTGTTGGCTTTAGGATGGACGTTAGGAGGTGACTTTAGGAG                                                                                                                                                                                                        <  1:571639/298‑1`  
                                                                                                                                       `GGTTAGACTTTAAGTCAATACTCTTTTTGATAAATTTTAAAGTAATCTTTTAAGTCTCTTTAAGTTGTCTCTCCTTATAGTGAGTTGTATTAATAAAGACAATGATTAAAGGTTATCACTTTATGGCTAACAACAAGGCGTCTTTAGGTCTG‑‑‑‑‑‑‑‑‑‑‑‑‑‑GTCTTTAGGTCTGGTCTTTATGTTTAAACTTTAAGATAGGCGTTGACTTGATGGGTCTTTAGGTGTAGGCTTTAGGTGTTGGCTTTAGGATGGACGTTAGGAGGTGACTTTAGGAGGATACTTTAGGAGACTGTAACAGATAGGGACAC                                                                                                                                                                       <  1:212166/301‑1`  
                                                                                                                                                                                                                                                               `taagagacagCAACAAGGCGTCGTTAGGTCTGGTCTTTATGTAG‑TGTCTTTAGGTCTGGTCTTTATGTTTAAACTTTAAGATAGGCGTTGACTTGATGGGTCTTTAGGTGTAGGCTTTAGGTGTTGGCTTTAGGATGGACGTTAGGAGGTGACTTTAGGAGGATACTTTAGGAGACTGTAACAGATAGGGACACAGAGAGACACTCAAGGTAACACCCAAAGACAAACCCAAGGGTCATCCCCAACCCTTAGGGAACCCTCAAGGTCAACCGAAGGTTGACTTTAGGTGATTGGCTGGGCT                                                            <  1:311269/291‑1`  
                                                                                                                                                                                                                                                                                       `ctcggagatgtgtataagagacagCTTTAGGTCTGGTCTTTATGTTTAAACTTTAAGATAGGCGTTGACTTGATGGGTCTTTAGGTGTAGGCTTTAGGTGTTGGCTTTAGGATGGACGTTAGGAGGTGACCTTAGGAGGATACTTTAGGAGACTGTAACAGATAGGGACACAGAGAGACACTCAAGGTAACACCCAAAGACAAACCCAAGGGTCATCCCCAACCCTTAGGGAACCCTCAAGGTCAACCGAAGGTTGACTTTAGGTGATTGGCTGGGCTTTAGGTACCCCCTATGGGGGAAC                                     <  1:298819/277‑1`  
                                                                                                                                                                                                                                                                                               `tgtgtataagagacagCTTTATGTCTGGTCTTTATGTTTAAACTTTAAGATAGGCGTTGACTTGATGGGTCTTTAGGTGTAGGCTTTAGGTGTTGGCTTTAGGATGGACGTTAGGAGGTGACTTTAGGAGGATACTTTAGGAGACTGTAACAGATAGGGACACAGAGAGACACTCAAGGTAACACCCAAAGACAAACCCAAGGGTCATCCCCAACCCTTAGGGAACCCTCAAGGTCAACCGAAGGTTGACTTTAGGTGATTGGCTGGGCTTTAGGTACCCCCTATGGGGGAACTTTAGGTC                             <  1:540693/285‑1`  
                                                                                                                                                                                                                                                                                               `ggtgttgaagagacagCTTTAGGTTTGGTCTTTATGTTTAATCTTTAAGATAGGCGTTGACTTGATGGGTCTTTAGGTGTAGGCTATAGGTGTTGGCTTTAGGATGGACGTTAGGAGGTGACTTTAGGAGGATACTTTAGGAGACTGTAACAGATAGGGACACAGAGAGACACTCAAGGTAACACCCAAAGACAAACCCAAGGGTCATCCCCAACCCTTAGGGAACCCTCAAGGTCAACCGAAGGTTGACTTTAGGTGATTGGCTGGGCTTTAGGTACCCCCTATGGGGGAACTTTAGGTC                             <  1:58476/285‑1`  
                                                                                                                                                                                                                                                                                               `ggtgtataagagacagCTTTAGGTCTGGTCTTTATGTTTAAACTTTAAGATAGGCGTTGACTTGATGGGTCTTTAGGTGTAGGCTTTAGGTGTTGGCTTTAGGATGGACGTTAGGAGGTGACTTTAGGAGGATACTTTAGGAGACTGTAACAGATAGGGACACAGAGAGACACTCAAGGTAACACCCAAAGACAAACCCAAGGGTCATCCCCAACCCTTAGGGAACCCTCAAGGTCAACCGAAGGTTGACTTTAGGTGATTGGCTGGGCTTTAGGTACCCCCTATGGGGGAACTTTAGGTC                             <  1:468631/285‑1`  
                                                                                                                                                                                                                                                                                               `ggctcggagatgtgtataagagacagGGTCTTTATGTTTAAACTTTAAGATAGGCGTTGACTTGATGTGTCTTTAGGTGTAGGCTTTAGGTGTTGGCTTTAGGATGTACGTTAGGAGGTGACTTTAGGAGGATACTTTAGGAGACTGTAACAGATAGGGACACAGAGAGACACTCAAGGTAACACCCAAAGACAAACCCAAGGGTCATCCCCAACCCTTAGGGAACCCTCAAGGTCAACCGAAGGTTGACTTTAGGTGATTGGCTGGGCTTTAGGTACCCCCTATGGGGGAACTTTAGGTC                             <  1:419820/275‑1`  
                                                                                                                                                                                                                                                                                                    `ataagagactTCTTTAGGTCTGGTCGTTATGTTTAAACTTTAAGATAGGCGTTGACTAGATGGGTCTTTAGGTGTAGGCTTTAGGTTTTGGCTTTAGGATGGACGTTAGGAGGTGACTTTAGGAGGATACTTTAGGAGACTGTAACAGATAGGGACACAGAGAGACACTCAAGGTAACACCCAAAGACAAACCCAAGGGTCATCCCCAACCCTTAGGGAACCCTCAAGGTCAACCGAAGGTTGACTTTAGGTGATTGGCTGGGCTTTAGGTACCCCCTATGGGGGAACTTTAGGTCCGTAC                        <  1:18502/291‑1`  
                                                                                                                                                                                                                                                                                                    `ataagagacagCTTTAGGTCTGGTCTTTATGTTTAAACTTTAAGATAGGCGTTGACTTGATGGGTCTTTAGGTGTAGGCTTTAGGTGTTGGCTTTAGGATGGACGTTAGGAGGTGACTTTAGGAGGATACTTTAGGAGACTGTAACAGATAGGGACACAGAGAGACACTCAAGGTAACACCCAAAGACAAACCCAAGGGTCATCCCCAACCCTTAGGGAACCCTCAAGGTCAACCGAAGGTTGACTTTAGGTGATTGGCTGGGCTTTAGGTACCCCCTATGGGGGAACTTTAGGTCCGTAC                        <  1:16594/290‑1`  
                                                                                                                                                                                                                                                                                                    `ataagagacagCTTTAGGTCTGGTCTTTATGTTTAAACTTTAAGATAGGCGTTGACTTGATGGGTCTTTAGGTGTAGGCTTTAGGTGTTGGCTTTAGGATGGACGTTAGGAGGTGACTTTAGGAGGATACTTTAGGAGACTGTAACAGATAGGGACACAGAGAGACACTCAAGGTAACACCCAAAGACAAACCCAAGGGTAATCCCCAACCCTTAGGGAACCCTCAAGGTCAACCGAAGGTTGACTTTAGGTGATTGGCTGGGCTTTAGGTACCCCCTATGGGGGAACTTTAGGTCCGTAC                        <  1:424347/290‑1`  
                                                                                                                                                                                                                                                                                                    `ataagagacagCTTTAGGTCTGGTCTTTATGTTTAAACTTTAAGATAGGCGTTGACTTGAGGGGTCTTTAGGTGTAGGCTTTAGGTGTTGGCTTTAGGATGGACGTTAGGAGGTGACTTTAGGAGGATACTTTAGGAGACTGTAACAGATAGGGACACATAGAGACACTCAAGGTAACACCCAAAGACAAACCCAAGGGTCATCCCCAACCCTTAGGGAACCCTCAAGGTCAACCGAAGGTTGACTTTAGGTGATTGGCTGGGCTTTAGGTACCCCCTATGGGGGAACTTTAGGTCCGTAC                        <  1:157785/290‑1`  
                                                                                                                                                                                                                                                                                                     `tggtcG‑TGTCTTTAGGTCTGGACTTTATGTTTAAACTTTAAGATAGGCGTTGACTTGATGGGTCTTTAGGTGAAGGCTTTATGTGTTGGCTTTAGGATGGACGTTAGGAGGTGACTTTAGGAGGATACTTTAGGAGACTGTAACAGATAGGGACACAGAGAGACACTCAAGGTAACACCCAAAGACAAACCCAAGGGTCATCCCCAACCCTTAGGGAACCCTCAAGGTCAACCGAAGGTTGACTTTAGGTGATTGGCTGGGCTTTAGGTACCCCCTATGGGGGAACTTTAGGTCCGTACAC                      <  1:389962/296‑1`  
                                                                                                                                                                                                                                                                                                      `tgttaaagagacagGGTCTGGTATTAAGGTTTAAACTTTAAGATAGGCGTTGAATTGATGGGTCGTTAGGTGTAGGATTTAGGTGTTGGCGTTAAGATGGACGTTAGGAGGTGAATTTAGGAGGATACTTTAGGAGACTGGAACAGATAGGGACACAGAGAGACACTCAAGGTAACAACCAAAGACAAAACCAAGGGTCATCCCCAACCCTTAGGGAACCCTCAAGGTCAACCGAAGGTTGACTTTAGGGGATTGGCTGGGCTTTAGGTACCCCCTATGGGGGAACTTTAGGTCCGTACAC                      <  1:643205/287‑1`  
                                                                                                                                                                                                                                                                                                      `tgtataagagacagtgttTGGTCTTTATGTTTAAACTTTAAGATAGGCGTTGACTTGATGGGTCTTTAGGTGTAGGCTTTAGGTGTTGGCTTTAGGATGGACGTTAGGAGGTGACTTTAGGAGGATACTTTAGGAGACTGTAACAGATAGGGACACAGAGAGACACTCAAGGTAACACCCAAAGACAAACCCAAGGGTCATCCCCAACCCTTAGGGAACCCTCAAGGTCAACCGAAGGTTGACTTTAGGTGATTGGCTGGGCTTTAGGTACCCCCTATGGGGGAACTTTAGGTCCGTACAC                      <  1:63466/283‑1`  
                                                                                                                                                                                                                                                                                                      `tgtataagagacagGGTCTGGTCTTTATGTTTAAACTTTAAGATAGGCGTTGACTTGATGGGTCTTTAGGTGTAGGCTTTAGGTGTTGGCTTTAGGATGGACGTTAGGAGGTGACTTTAGGAGGATACTTTAGGAGACTGTAACAGATAGGGACACAGAGAGACACTCAAGGTAACACCCAAAGACAAACCCAAGGGTCATCCCCAACCCTTAGGGAACCCTCAAGGTCAACCGAAGGTTGACTTTAGGTGATTGGCTGGGCTTTAGGTACCCCCTATGGGGGAACTTTAGGTCCGTACAC                      <  1:262125/287‑1`  
                                                                                                                                                                                                                                                                                                      `tgtataagagacagGGTCTGGTCTTTATGTTTAAACTTTAAGATAGGCGTTGACTCGATGGGTCTTTAGGTGTAGGCTTTAGGTGTTGGCTTTAGGATGGACGTTAGGAGGTGACTTTAGGAGGATACTTTAGGAGACTGTAACAGATAGGGACACAGAGAGACACTCAAGGTAACACCCAAAGACAAACCCAAGGGTCATCCCCAACCCTTAGGGAACCCTCAAGGTCAACCGAAGGTTGACTTTAGGTGATTGGCTGGGCTTTAGGTACCCCCTATGGGGGAACTTTAGGTCCGTACAC                      <  1:252251/287‑1`  
                                                                                                                                                                                                                                                                                                      `tgtataagagacagGGTCTGGTCTTGATGTTTAAACTTTAAGATAGGCGTTGACTTGATGGGTCTTTAGGTGTAGGCTTTAGGTGTTGGCTTTAGGATGGACGTTAGGAGGTGACTTTAGGAGGATACTTTAGGAGACTGTAACAGATAGGGACACAGAGAGACACTCAAGGTAACACCCAAAGACAAACCCAAGGGTCATCCCCAACCCTTAGGGAACCCTCAAGGTCAACCGAAGGTTGACTTTAGGTGATTGGCTGGGCTTTAGGTACCCCCTATGGGGGAACTTTAGGTCCGTACAC                      <  1:396033/287‑1`  
                                                                                                                                                                                                                                                                                                      `gtataagagacagAGGTCTGGTCTTTATGTTTAAACTTTAAGATAGGCGTTGACTTGATGGGTCTTTAGGTGTAGGCTTTAGGTGTTGGCTTTAGGATGGACGTTAGGAGGTGACTTTAGGAGGATACTTTAGGAGACTGTAACAGATAGGGACACAGAGAGACACTCAAGGTAACACCCAAAGACAAACCCAAGGGTCATCCCCAACCCTTAGGGAACCCTCAAGGTCAACCGAAGGTTGACTTTAGGTGATTGGCTGGGCTTTAGGTACCCCCTATGGGGGAACTTTAGGTCCGTACAC                      <  1:566014/288‑1`  
                                                                                                                                                                                                                                                                                                      `gagatgtgtataagagacaGGTCTTTATGTTTAAACTTTAAGATAGGCGTTGACTTGATGGGTCTTTAGGTGTAGGCTTTAGGTGTTGGCTTTAGGATGGGCGTTAGGAGGTGACTTTAGGAGGATACTTTAGGAGACTGTAACAGATAGGGACACAGAGAGACACTCAAGGTAACACCCAAAGACAAACCCAAGGGTCATCCCCAACCCTTAGGGAACCCTCAAGGTCAACCGAAGGTTGACTTTAGGTGATTGGCTGGGCTTTAGGTACCCCCTATGGGGGAACTTTAGGTCCGTACAC                      <  1:579797/282‑1`  
                                                                                                                                                                                                                                                                                                      `gagatgtgtataagagacaGGTCTTTATGTTTAAACTTTAAGATAGGCGTTGACTTGATGGGTCTTTAGGTGTAGGCTTTAGGTGTTGGCTTTAGGATGGACGTTAGGAGGTGACTTTAGGAGGATACTTTAGGAGACTGTAACAGATAGGGACACAGAGAGACACTCAAGGTAACACCCAAAGACAAACCCAAGGGTCATCCCCAACCCTTAGGGAACCCTCAAGGTCAACCGAAGGTTGACTTTAGGTGATTGGCTGGGCTTTAGGTACCCCCTATGGGGGAACTTTAGGTCCGTACAC                      <  1:118528/282‑1`  
                                                                                                                                                                                                                                                                                                      `atgtctTGTCTTTAGGTCTGGTCTGTATGTTTAAACTTTAAGATAGGCTGTGACTTGATGGGTCTTTAGGTGTAGGCTTTAGGTGTTGTCTTTAGGATGGACGTTAGGAGGTGACTTTATGAGGATACTGTAGGAGACTGTAACAGATAGGGACACAGAGAGACACTCAAGGTAACACCCAAAGACAAACCCAAGGGTCATCCCCAACCCTTAGGGAACCCTCAAGGTCAACCGAAGGTTGACTTTAGGTGATTGGCTGGGCTTTAGGTACCCCCTATGGGGGAACTTTAGGTCCGTACAC                      <  1:322763/295‑1`  
                                                                                                                                                                                                                                                                                                      `aagagacagCTTTAGGTCTTGTCTTTATGTTTAAACTTTAAGATAGGCGTTGACTTGATGGGTCTTTAGGTGTAAGCTTTAGGTGTTGGCTTTAGGATGGACGTTAGGAGGTGACTTTAGGAGGATACTTTAGGAGACTGTAACAGATAGGGACACAGAGAGACACTCAAGGTAACACCCAAAGGCAAACCCAAGGGTCATCCCCAACCCTTAGGGAACCCTCAAGGTCAACCGAAGGTGGACTTTAGGTGATTGGCTGGGCTTTAGGTACCCCCTATGGGGGAACTTTAGGTCCGTACAC                      <  1:3416/292‑1`  
                                                                                                                                                                                                                                                                                                      `aagagacagCTTTAGGTCTGGTCTTTATGTTTAAACTTTAAGATAGGCGTTGACTTGATTGGTCTTTAGGTGTAGGCTTTAGGTGTTGGCTTTAGGATGGACGTTAGGAGGTGACTTTAGGAGGATACTTTAGGAGACTGTAACAGATAGGGACACAGAGAGACACTCAAGGTAACACCCAAAGACAAACCCAAGGGTCATCCCCAACCCTTAGGGAACCCTCAAGGTCAACCGAAGGTTGACTTTAGGTGATTGGCTGGGCTTTAGGTACCCCCTATGGGGGAACTTTAGGTCCGTACAC                      <  1:560801/292‑1`  
                                                                                                                                                                                                                                                                                                      `aagagacagCTTTAGGTCTGGTCTTTATGTTTAAACTTTAAGATAGGCGTTGACTTGATGGGTCTTTAGGTGTAGGCTTTAGGTGTTGGCTTTAGGATGGACGTTAGGAGGTGACTTTAGGAGGATACTTTAGGAGACTGTAACAGATAGGGACACAGAGAGACACTCAAGGTAACACCCAAAGACAAACCCAAGGGTCATCCCCAACCCTTAGGGAACCCTCAAGGTCAACCGAAGGTTGACTTTAGGTGATTGGCTGGGCTTTAGGTACCCCCTATGGGGGAACTTTAGGTCCGTACAC                      <  1:509413/292‑1`  
                                                                                                                                                                                                                                                                                                             `GTCTTTAGGTCTGGTCTTTATGTTTAAACTTTAAGATAGGCGTTGACTTGATGGGTCTTTAGGTGTAGGCTTTAGGTGTTGTCTTTAGGATGGACGTTAGGAGGTGACTTTAGGAGGATACTTTAGGAGACTGTAACGGATAGGGACACAGAGAGACACTCAAGGTAACACCCAAAGACAAACCCAAGGGTAATCCCCAACCCTTAGGGAACCCTCAAGGTCAACCGAAGGTTGACTTTAGGTGATTGGCTGGGCTTTAGGTACCCCCTATGGGGGAAATTTAGGTCCtgtctcttataca               >  1:295543/1‑288`  
                                                                                                                                                                                                                                                                                                             `GTCTTTAGGTCTGGTCTTTATGTTTAAACTTTAAGATAGGCGTTGACTTGATGGGTCTTTAGGTGTAGGCTTTAGGTGTTGGCTTTAGGTTGGACGTTAGGAGGTGACTTTAGGAGGATACTTTAGGAGACTGTAACAGATAGGGACACAGAGAGACACTCAAGGTAACACCCAAAGACAAATCCAAGGGTCATCCCCAACCCTTAGGGAACCCTCAAGGTCAACCGAAGGTTGACTTTAGGTGATTGGCTGGGCTTTAGGTACCCCCTATGGGGGAACTTTAGTTCCGTACACctgtctc               >  1:45177/1‑294`  
                                                                                                                                                                                                                                                                                                             `GTCTTTAGGTCTGGTCTTTATGTTTAAACTTTAAGATAGGCGTTGACTTGATGGGTCTTTAGGTGTAGGCTTTAGGTGTTGGCTTTAGGATGGACGTTAGGAGGTGACTTTAGGAGGTTACTTTAGGAGACTGTAACATATAGGGACACAGAGAGACACACAAGGTAACACCCAAAGACAAACCCAAGGGTCATCCCCAACCCTTAGGGAACCCCCAAGGTCAACCGAAGGTTGACTTTCGGTGATTGGCTGGGCTTTAGCTACCCCCACTGGGGGACATTTAGGTCCGTACACctgcccc               >  1:639085/1‑294`  
                                                                                                                                                                                                                                                                                                             `GTCTTTAGGTCTGGTCTTTATGTTTAAACTTTAAGATAGGCGTTGACTTGATGGGTCTTTAGGTGTAGGCTTTAGGTGTTGGCTTTAGGATGGACGTTAGGAGGTGACTTTAGGAGGATACTTTAGGAGACTGTAACATATAGGGACACAGAGAGACACTCAAGGTAACACCCAAAGACAAACCCAAGGGTCATCCCCAACCCTTAGGGAACCCTCAAGGTCAACCGAAGGTTGACTTTAGGTGATTGGCTGGGCTTTAGGTACCCCCTATGGGGGAACTTTAGGTCCGTACACctgtctc               >  1:429680/1‑294`  
                                                                                                                                                                                                                                                                                                             `GTCTTTAGGTCTGGTCTTTATGTTTAAACTTTAAGATAGGCGTTGACTTGATGGGTCTTTAGGTGTAGGCTTTAGGTGTTGGCTTTAGGATGGACGTTAGGAGGTGACTTTAGGAGGATACTTTAGGAGACTGTAACAGATAGGGACACAGAGAGACACTCAAGGTAATACCCAAAGACAAACCCAAGGGTCATCACCAACCCTTAGGGAACCCTCAAGGTCAACCGAAGGTTGACTTTAGGTGATTGGCTGGGCTTTAGGTACCCCCTCTGGGGGAACTTTAGGTCCtgtctcttataca               >  1:138842/1‑288`  
                                                                                                                                                                                                                                                                                                             `GTCTTTAGGTCTGGTCTTTATGTTTAAACTTTAAGATAGGCGTTGACTTGATGGGTCTTTAGGTGTAGGCTTTAGGTGTTGGCTTTAGGATGGACGTTAGGAGGTGACTTTAGGAGGATACTTTAGGAGACTGTAACAGATAGGGACACAGAGAGACACTCAAGGTAACACCCAAAGACAAACCCACGGGTCATCCCCAACCCTTAGGGAACCCTCAAGGTCAACCGAAGGTTGACTTTAGGTTATTGGCTGGGCTTTAGGTACCCCCTATGGGGGAACTTTAGGTCCGTACctgtctctt               >  1:490631/1‑292`  
                                                                                                                                                                                                                                                                                                               `CTTTAGGTCTGGTCTTTATGTTTAAACTTTAAGATAGGCGTTGACTTGATGGGTCTTTAGGTTTAGGCTTTTGGCGTTGGCTTTAGGATGGACGTTAGGAGGTGACTTTAGTAGGATACTTTAGGAGACTGTAACAGATAGGGACACAGAGAGACACTCAAGTTAATAACCACAGACAAACCCAAGGGTCATCCCCACCCCTTAGAGAACCCTCACGGTCAACCGAAGGTTGACTTTAGGTGCTTGGCTGGGCTTTAGGTACCCCCTAGGGGGGGACTTTAGGTCCGTACAacagtctctt             >  1:360676/1‑291`  
                                                                                                                                                                                                                                                                                                               `CTTTAGGTCTGGTCTTTATGTTTAAACTTTAAGATAGGCGTTGACTTGATGGGTCTTTAGGTGTAGGCTTTAGGTGTTGGCTTTAGGCTGGACGTTAGGAGGTGACTTTAGGAGGATACTTTAGGAGACTGTAACAGATAGGGACACAGAGAGACACTCAAGGTAACACCCAAAGACAAACCCACGGGTCATCCCCAACCCTTAGGGAACCCTCAAGGCCAACCGAAGGTTGACTTTAGGTGATTGGCTGGGCTTTCGGTCCCCCCTATGGGGGACCTTTAGGTCCGTACACctgtctctt             >  1:225414/1‑292`  
                                                                                                                                                                                                                                                                                                               `CTTTAGGTCTGGTCTTTATGTTTAAACTTTAAGATAGGCGTTGACTTGATGGGTCTTTAGGTGTAGGCTTTAGGTGTTGGCTTTAGGATGGACGTTAGGAGGTGACTTTAGGAGGATACTTTAGGAGACTGTAACAGATAGGGACACAGAGAGACACTCTAGGTAACACCCAAAGACAAACCCAAGGGTCATCCCCAACCCTTAGGGAACCCTCAAGGTCAACCGAAGGTTGACTTTAGGTGATTGGCT‑GGCTTTAGGTACCCCCTATGGGGGAACTTTATGTCCGTACACctgtctcttt            >  1:151542/1‑291`  
                                                                                                                                                                                                                                                                                                               `CTTTAGGTCTGGTCTTTATGTTTAAACTTTAAGATAGGCGTTGACTTGATGGGTCTTTAGGTGTAGGCTTTAGGTGTTGGCTTTAGGATGGACGTTAGGAGGTGACTTTAGGAGGATACTTTAGGAGACTGTAACAGATAGGGACACAGAGAGACACTCAAGGTAACACCCAAAGACAAACCCATGGGTCATCCCCAACCCTTAGGGAACCCTCAAGGTCAACCGAAGGTTGTCTTTAGGTGATTGGCTGGGCTTTAGGTACCCCCTATGGGGGAACTTTAGGTCCGTACctgtctcttat             >  1:330316/1‑290`  
                                                                                                                                                                                                                                                                                                               `CTTTAGGTCTGGTCTTTATGTTTAAACTTTAAGATAGGCGTTGACTTGATGGGTCTTTAGGTGTAGGCTTTAGGTGTTGGCTTTAGGATGGACGTTAGGAGGTGACTTTAGGAGGATACTTTAGGAGACTGTAACAGATAGGGACACAGAGAGACACTCAAGGTAACACCCAAAGACAAACCCAAGGGTCATCCCCAACCCTTAGGGAACCCTCAAGGTCAACCGAAGGTTGACTTTAGGTGATTGGCTGGGCTTTAGGTACCCCCTATGGGGGAACTTTAGGTCCtgtctcttatacaca             >  1:121666/1‑286`  
                                                                                                                                                                                                                                                                                                               `CTTTAGGTCTGGTCTTTATGTTTAAACTTTAAGATAGGCGTTGACTTGATGGGTCTTTAGGTGTAGGCTTTAGGTGTTGGCTTTAGGATGGACGTTAGGAGGTGACTTTAGGAGGATACTTTAGGAGACTGTAACAGATAGGGACACAGAGAGACACTCAAGGTAACACCCAAAGACAAACCCAAGGGTCATCCCCAACCCTTAGGGAACCCTCAAGGTCAACCGAAGGTTGACTTTAGGTGATTGGCTGGGCTTTAGGTACCCCCTATGGGGGAACTTTAGGTCCGTACACatgtctctt             >  1:661534/1‑292`  
                                                                                                                                                                                                                                                                                                               `CTTTAGGTCTGGTCTTTATGTTTAAACTTTAAGATAGGCGTTGACTTGATGGGTCTTTAGGTGTAGGCTTTAGGTGTTGGCTTTAGGATGGACGTTAGGAGGTGACTTTAGGAGGATACTTTAGGAGACTGTAACAGATAGGGACACAGAGAGACACTCAAGGTAACACCCAAAGACAAACCCAAGGGTCATCCCCAACCCTTAGGGAACCCTCAAGGTCAACCGAAGGTTGACTTTAGGTGATTGGCTGGGCTTTAGGTACCCCCTATGGGGGAACTTTAGGTACGTACACctgtctctt             >  1:272467/1‑292`  
                                                                                                                                                                                                                                                                                                               `CTTTAGGTCTGGTCTTTATGTTTAAACTTTAAGATAGGCGTTGACTTGATGGGTCTTTAGGTGTAGGCTTTAGGTGTTGGCTTTAGGATGGACGTTAGGAGGTGACTTTAGGAGGATACTTTAGGAGACTGTAACAGATAGGGACACAGAGAGACACTCAAGGTAACACCCAAAGACAAACCCAAGGGTCATCCCAAACCCTTAGGGAACCCTCAAGGTCAACCGAAGGTTGACTTTAGGTGATTGGCTGGGCTTTAGGTACCCCCTATGGGGGAACTTTAGGTCCGTACACctgtctctt             >  1:457237/1‑292`  
                                                                                                                                                                                                                                                                                                                    `GGTCTGGTCTTTATGTTTAAACTTTATGATAGGCGTTGACTTGATGGGTCTTTAGGTGTAGGCTTTAGGTTTTGGCTTTAGGATGGACGTTAGGAGGTGACTTTAGGAGGATACTTTAGGAGACTGTAACAGATAGGGACACAGAGAGACACTCAAGGTAACACCCAAAGACAAACCCAAGGGTCATCACCAACCCTTAGGGAACACTCAAGGTCAACCGACGGTTGACTTTCGGTGATTGGCTGGGCTTTAGGTACCCCCTATAGGGGAACTTTAGGTCCtttctcttatccacttctcc        >  1:651807/1‑281`  
                                                                                                                                                                                                                                                                                                                    `GGTCTGGTCTTTATGTTTAAACTTTAAGATAGGCGTTGACTTGATGGGTCTTTAGGTGTAGGCTTTAGGTGTTGGCTTTAGGATGGACGTTAGGAGGTGACTTTAGGAGGATACTTTAGGAGACTGTAACAGATAGGGACACAGAGAGACACTCAAGGTAACACCCAAAGACAAACCCAAGGGTCATCCCCAACCCTTAGGGAACCCTCAAGGTCAACCGAAGGTTGACTTTAGGTGATTGGCTGGGCTTTAGGTACCCCCTATGGGGGAACTTTAGGTCCGTACACctgtctcttataca        >  1:596208/1‑287`  
                                                                                                                                                                                                                                                                                                                    `GGTCTGGTCTTTATGTTTAAACTTTAAGATAGGCGTTGACTTGATGGGTCTTTAGGTGTAGGCTTTAGGTGTTGGCTTTAGGATGGACGTTAGGAGGTGACTTTAGGAGGATACTTTAGGAGACTGTAACAGATAGGGACACAGAGAGACACTCAAGGTAACACCCAAAGACAAACCCAAGGGTCATCCCCAACCATTAGGGAACCCTCAAGGTCAACCGAAGGTTGAATTTAGGCGATTGGCTGGGCTTTAGTTACCCCCTATGGGGGAACTTTAGGTCAGTACACctgtctcttataca        >  1:63106/1‑287`  
                                                                                                                                                                                                                                                                                                                         `GGTCTTTATGTTTAAACTTTAAGATAGGCGTTGACTTGATGGGTCTTTAGGTGTAGGCTTTAGGTGTTGGCTTTAGGTTGGACGTTAGGAGGTGACTTTAGGAGGATACTTTAGGAGACTGTAACAGATAGGGACACAGAGAGACACTCAAGGTAACACCCAAAGACAAACCCAAGGGTCATCCCCAACCCTTAGGGAACCCTCAAGGTCAACCGAAGGTTGACTTTAGGTGATTGGCTGGGCTTTAGGTACCCCCTATGGGGGAACTTTAGGTCCGTACACctgtctcttctacccatct   >  1:386320/1‑282`  
                                                                                                                                                                                                                                                                                                                         `GGTCTTTATGTTTAAACTTTAAGATAGGCGTTGACTTGATGGGTCTTTAGGTGTAGGCTTTAGGTGTTGGCTTTAGGATGGACGTTAGGAGGTGACTTTAGGAGGATACTTTAGGAGACTGTAACAGATAGGGACACAGAGAGACACTCAAGGTAACACCCAAAGACAAACCCAAGGGTCATCCCCAACCCTTAGGGAACCCTCAAGGTCAACCGAAGGTTGACTTTAGGTGATTGGCTGGGCTTTAGGTACCCCCTATGGGGGAACTTTAGGTCCGTACACctgtctcttatacacatct   >  1:348599/1‑282`  
                                                                                                                                                                                                                                                                                                                          `GTCTTTATGTTTAAACTTTAAGATAGGCGTTGACTTGATGGGTCTTTAGGTGTAGGCTTTAGGTGTTGGCTTTAGGATGGACGTTAGGAGGTGACTTTAGGAGGATACTTTAGGCGACTGTAACAGATAGGGACACAGAGAGACACTCAAGGTAACACCCCAAGACAAACCCAAGGGTCATCCCCAACCCTTAGGGAACCCTCAAGGTCAACCGAAGGTTGACTTTAGGTGATTGGCTGGGCTTTAGGTACCCCCTATGGTGGAACTTTAGGTCCGTACACctgtctcttatacacatctc  >  1:203560/1‑281`  
                                                                                                                                                                                                                                                                                                                          `GTCTTTATGTTTAAACTTTAAGATAGGCGTTGACTTGATGGGTCTTTAGGTGTAGGCTTTAGGTGTTGGCTTTAGGATGGACGTTAGGAGGTGACTTTAGGAGGATACTTTAGGAGACTGTAACAGATAGGGACACAGAGAGACACTCAAGGTAACACCCAAAGACAAACCCAAGGGTCATCCCCAACCCTTAGGGAACCCTCAAGGTCAACCGAAGGTTGACTTTAGGTGATTGGCTGGGCTTTAGGTACCCCCTATGGGGGAACTTTAGGTCCGTACctgtctcttatacaaatctccg  >  1:398873/1‑279`  

                                                                                                                                                                                                                                                                                                                                                                                                                                                                                                                                                                                                                                         
`TACTGCATGTTACTTCATGTTGTCAATACCTGTTTTTCGTGCGACTTATCAGGCTGTCTACTTATCCGGTTGACCCCGGTGTCGATTGGGATGGCTATTCGCCGTGTCCCTCTCGATGGCTGTAAGTATCCTATAGGTTAGACTTTAAGTCAATACTCTTTTTGATAAATTTTAAATTAATCTTTTAAGTCTCTTTAAGTTGTCTCTCCTTATAGTGAGTTGTATTAATAAAGACAATGATTAAAGGTTATCACTTTATGGCTAACAACAAGGCGTCTTTAGGTCTGGTCTTTATGTAG‑TGTCTTTAGGTCTGGTCTTTA‑‑‑‑‑‑‑‑‑‑‑‑‑‑‑‑‑‑‑‑‑‑‑‑‑‑‑‑‑‑‑‑‑‑‑‑‑‑‑‑‑‑‑‑‑‑‑‑‑‑‑‑‑‑‑‑‑‑‑‑‑‑‑‑‑‑‑‑‑‑‑‑‑‑‑‑‑‑‑‑‑‑‑‑‑‑‑‑‑‑‑‑‑‑‑‑‑‑‑‑‑‑‑‑‑‑‑‑‑‑‑‑‑‑‑‑‑‑‑‑‑‑‑‑‑‑‑‑‑‑‑‑‑‑‑‑‑‑‑‑‑‑‑‑‑‑‑‑‑‑‑‑‑‑‑‑‑‑‑‑‑‑‑‑‑‑‑‑‑‑‑‑‑‑‑‑‑‑‑‑‑‑‑‑‑‑‑‑‑‑‑‑‑‑‑‑‑‑‑‑‑‑‑‑‑‑‑‑‑‑‑‑‑‑‑‑‑‑‑‑‑‑‑‑‑‑‑‑‑‑‑‑‑‑‑‑‑‑‑‑‑‑‑‑‑‑‑‑‑‑‑‑‑‑‑‑‑‑‑‑‑‑‑‑‑‑‑‑‑‑‑‑‑‑‑‑‑‑‑‑‑‑‑‑‑‑‑‑‑‑‑‑‑‑  <  NC_001604.1/614‑295`  
`‑‑‑‑‑‑‑‑‑‑‑‑‑‑‑‑‑‑‑‑‑‑‑‑‑‑‑‑‑‑‑‑‑‑‑‑‑‑‑‑‑‑‑‑‑‑‑‑‑‑‑‑‑‑‑‑‑‑‑‑‑‑‑‑‑‑‑‑‑‑‑‑‑‑‑‑‑‑‑‑‑‑‑‑‑‑‑‑‑‑‑‑‑‑‑‑‑‑‑‑‑‑‑‑‑‑‑‑‑‑‑‑‑‑‑‑‑‑‑‑‑‑‑‑‑‑‑‑‑‑‑‑‑‑‑‑‑‑‑‑‑‑‑‑‑‑‑‑‑‑‑‑‑‑‑‑‑‑‑‑‑‑‑‑‑‑‑‑‑‑‑‑‑‑‑‑‑‑‑‑‑‑‑‑‑‑‑‑‑‑‑‑‑‑‑‑‑‑‑‑‑‑‑‑‑‑‑‑‑‑‑‑‑‑‑‑‑‑‑‑‑‑‑‑‑‑‑‑‑‑‑‑‑‑‑‑‑‑‑‑‑‑‑‑‑‑‑‑‑‑‑‑‑‑‑‑‑‑‑‑‑‑‑‑‑‑‑‑‑‑‑‑‑‑‑‑‑‑‑‑‑‑‑‑‑‑‑‑‑‑‑‑‑‑‑‑‑‑‑‑‑gtctttaggtctggtctttaTGTTTAAACTTTAAGATAGGCGTTGACTTGATGGGTCTTTAGGTGTAGGCTTTAGGTGTTGGCTTTAGGATGGACGTTAGGAGGTGACTTTAGGAGGATACTTTAGGAGACTGTAACAGATAGGGACACAGAGAGACACTCAAGGTAACACCCAAAGACAAACCCAAGGGTCATCCCCAACCCTTAGGGAACCCTCAAGGTCAACCGAAGGTTGACTTTAGGTGATTGGCTGGGCTTTAGGTACCCCCTATGGGGGAACTTTAGGTCCGTACACTGTGAGA‑‑‑‑‑‑‑‑‑‑‑‑‑  <  NC_001604.1/281‑1`

  

Alignment Legend

Aligned base mismatch/match (shaded by quality score): `ATCG/ATCG` `< 3 ≤` `ATCG/ATCG` `< 10 ≤` `ATCG/ATCG` `< 21 ≤` `ATCG/ATCG` `< 34 ≤` `ATCG/ATCG` `< 38 ≤` `ATCG/ATCG`

Unaligned base: `atcg`    Masked matching base: `atcg`    Alignment gap: `‑`    Deleted base: `‑`

Reads not counted as support for junction

read\_name Not counted due to insufficient overlap past the breakpoint.

read\_name Not counted due to not crossing MOB target site duplication.
